# Competition in the Segregation Mechanism of Granular Flow Within a 2D Rotating Drum Based on Magnetic Positioning Technology

**DOI:** 10.3390/s26061741

**Published:** 2026-03-10

**Authors:** Rong Pan, Zhi-Peng Chi, Yi-Ming Li, Ran Li, Hui Yang

**Affiliations:** 1 School of Optical-Electrical and Computer Engineering, University of Shanghai for Science and Technology, Shanghai 200093, China; 2 College of Medical Instrumentation, Shanghai University of Medicine and Health Sciences, Shanghai 201318, China

**Keywords:** magnetic positioning, particle segregation, dynamic tracking, competition mechanism

## Abstract

Accurate monitoring of internal particle motion in dense granular flows remains a significant challenge across various fields, ranging from geophysics to industrial processes. To address the limitations of existing observational techniques, this study presents a novel high-precision magnetic array positioning system based on magnetic dipole theory for dynamically tracking individual particles within opaque granular media. The system integrates an array of nine magnetic sensors with a hybrid optimization algorithm that combines Particle Swarm Optimization (PSO) and gradient-based local refinement, achieving a dynamic positioning accuracy within the maximum measurable range, with a maximum dynamic error of 2.5 ± 0.5 mm and a trajectory continuity exceeding 99%. Deployed in a quasi-two-dimensional rotating drum, the system enables detailed investigation of particle segregation mechanisms. Reconstruction and analysis of the trajectories of a high-density intruder (magnetic bead) allow quantification of the competition among segregation mechanisms through the Froude number. The results reveal three distinct motion phases with increasing rotational speed: a gravity-dominated percolation stage, a transitional collision–diffusion competition stage, and a centrifugal diffusion-dominated stage. Each phase exhibits unique kinematic signatures governed by the interplay of inertial, gravitational, and contact forces. This work not only establishes a robust and accurate sensor-based method for internal granular flow monitoring but also provides new mechanistic insights into segregation dynamics, with implications for understanding geological hazards such as debris flows.

## 1. Introduction

Geological hazards such as debris flows and landslides perennially occur in semi-arid mountainous regions or plateau glacial zones [1,2,3], and their disaster mechanisms and development prediction have attracted widespread scholarly attention in recent years. During the disaster process, due to the different properties (such as size and density) of internal particles, sorting becomes inevitable, which leads to the further development of disasters. In addition, the flow state of particles transitions from a dense regime to a more diffuse regime in the development process, exhibiting different kinematic characteristics. This process is essentially an entropy-increasing phenomenon of particle flow motion [4], which is a competitive process of a sorting mechanism developing from order to disorder, indicating that there is a close connection between flow velocity and the sorting mechanism [5]. However, limited by current observation techniques, the present stage is still dominated by simulations. An effective observation technology for internal particle motion trajectory is of great significance to analyze the competition relationship between sorting mechanisms.

The quasi-2D rotating drum is a standard experimental platform for studying free-surface flow due to its stability and periodicity [6,7]. While pioneering work by Savage, Ottino, and Gray established governing equations for advection and diffusion [8,9,10,11,12], quantifying these mechanisms experimentally remains difficult. In 2015, C.C. Liao proposed the use of a shape index to quantify the sorting morphology of particles in the rotary table. It was found that the shape index decreased monotonously when the rotating speed increased from 0.6 to 1.4 rpm, and the patch took longer to reach the steady state [13]. Experiments by Hou et al. at higher rotational speeds employed the Lacey index to quantify the steady-state pattern evolution. They observed that while surface velocities within the flow layer monotonically increased from 4 to 80 rpm [14], the time required for pattern stabilization did not decrease monotonically. This indicates differing sensitivities of sorting mechanisms to flow velocity and suggests competitive interactions between mechanisms. Within disc systems, the particle bed layer exhibits characteristic ‘tumbling motion’ in the low Froude number region (10–20 rpm), representing a critical state for investigating particle mixing mechanisms [15]. In 2005, Jain et al. studied the effects of particle size ratio, density ratio, and rotational speed on particle separation within SD systems (size-density), concluding that particle characteristics determine separation trends while rotational speed dictates separation extent [16,17]. Gray and Thornton established the fundamental dependence of segregation on particle size ratios, providing a general theoretical framework for particle segregation velocities [18]. Duan later included particle concentration as a parameter in their separation velocity model in the context of bounded heap flows [19]. While the convective diffusion equation holds clear physical significance at the macroscopic level, quantitative three-dimensional measurements of particle concentration remain challenging. Consequently, we seek a three-dimensional positioning technique to track individual particles, thereby elucidating their microscopic motion mechanisms and advancing the evolution of motion control models towards constitutive relationships.

Currently, traditional observation techniques for granular materials primarily focus on stress measurement and motion measurement [20,21]. Stress measurement, operating within the framework of approximate constitutive relationships, struggles to accurately reflect the internal motion characteristics of granular flows. Motion measurement, predominantly employing superficial observation methods such as imaging and ultrasound [22,23], fails to capture internal information of granular flows. As an emerging technology, magnetic positioning requires only the deployment of a magnetic sensor array to determine the position of a magnetic source. It offers higher positioning accuracy than GNSS/GPS within small-scale environments [24]. In 1821, Ampère proposed the magnetic dipole theory [25]. Building upon this, the Nara team developed a magnetic positioning system method [26]. Experimental studies have demonstrated that magnetic positioning systems can achieve higher sampling rates and greater accuracy than Ultra-Wideband (UWB) and inertial navigation systems in certain scenarios [27]. However, the core performance of magnetic positioning technology is highly dependent on the stability of the ambient magnetic field [28], an inherent limitation difficult to circumvent in complex environments. Particularly in dynamic positioning scenarios, disturbances caused by target motion persistently undermine positioning stability [29]. Furthermore, the effective operational range of magnetic positioning is constrained by the decay characteristics of magnetic signals. When the positioning distance exceeds a preset threshold, the signal-to-noise ratio drops sharply, leading to a cliff-like decline in accuracy, thus failing to meet the requirements for continuous dynamic positioning over large areas [30]. In response to these challenges, current research has undertaken targeted optimizations. For instance, Lv et al. [31] proposed a Kalman filter-based algorithm for suppressing magnetic interference, which achieves dynamic compensation of disturbance signals by modeling environmental interference. However, this method shows limited adaptability to sudden strong disturbances, and its model parameters require manual tuning for specific scenarios, lacking generalization capability. Meanwhile, Zhang’s team [32] attempted to integrate magnetic and WiFi signals to create a multi-source positioning system, leveraging WiFi to extend coverage. Nevertheless, the disparity in sampling frequencies between the two signals introduces time synchronization errors during data fusion, ultimately compromising positioning accuracy. Therefore, enhancing local fine-grained positioning to reconstruct continuous and complete trajectories represents a key focus in current magnetic positioning research.

In this paper, a high-precision magnetic array positioning technology based on magnetic dipole theory is proposed to realize the process monitoring and motion state of particle motion trajectory in three-dimensional space. The dynamic positioning accuracy of less than 3 mm is achieved, which effectively overcomes the shortcomings of traditional methods in sampling rate and positioning accuracy. This positioning technology was used in a quasi-2D turntable with 50% filling degree and a rotational speed range of 10–20 rpm [19] to explore the effect of rotational speed on the particle motion pattern and deepen people’s further understanding of the sorting competition mechanism. This study not only provides a new and reliable trajectory capture technical means for the study of particle motion in the drum, but also realizes the multi-scale observation from microscopic individual behavior to macroscopic system dynamics, which lays an experimental and theoretical foundation for the subsequent construction of more accurate particle motion models.

The remainder of this paper is structured as follows. Section 2 details the experimental setup and the magnetic positioning system. Section 3 presents the experimental results and discusses the underlying particle segregation mechanisms. Section 4 concludes the study and outlines directions for future work.

## 2. Materials and Methods

### 2.1. Experimental Design

**Figure 1:**
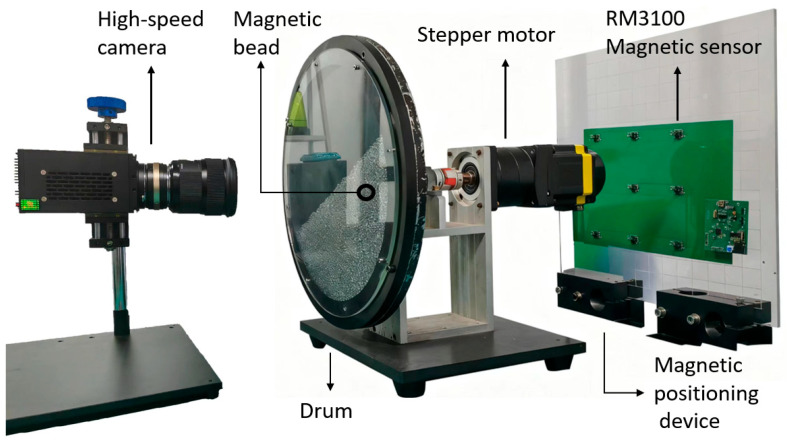
Experimental setup of the system.

The experimental device in Figure 1 includes a high-speed camera (Revealer, CHN), a turntable system (Customization, CHN) and a magnetic positioning device (Shenzhen JLC Technology Group Co., Ltd. CHN). The inner wall of the drum is 29 cm in diameter and 1 cm in thickness. Inside the drum is a spherical glass particle with a particle density of 2500 kg/m^3^ and a diameter of 3 mm, and the particle filling degree is 50%. A magnetic bead of the same particle size, with a density of 7500 kg/m^3^, is placed inside the particle group. The magnetic positioning device is used to detect the magnetic bead position inside the particle.

The device parameters are shown in Table 1.

### 2.2. The Magnetic Positioning System

**Figure 2:**
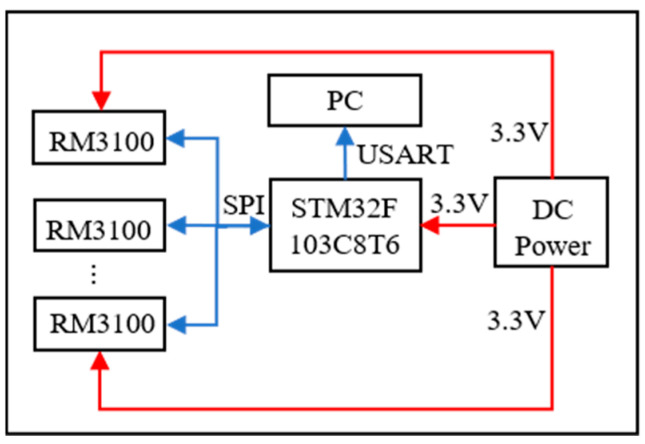
Working principle diagram of magnetic positioning system.

The magnetic positioning system is a magnetic array positioning system composed of nine magnetic sensors. It determines the position of the permanent magnet in the space by the principle of magnetic dipole. In order to obtain more accurate sensor sensitivity coefficients and related parameters such as position and direction, a calibration experiment needs to be conducted on the system in advance. During the calibration experiment, a permanent magnet with a diameter of 8 mm was selected, and its magnetization is 10.8–11.2 kA/m. Figure 1 shows a 3 × 3 matrix array consisting of 9 RM3100 magnetic sensors (Witmotio, CHN) integrated at the position where the measurement plate is fixed, with horizontal and vertical spacing of 80 mm. The measurement plate is fixed on a grid acrylic plate to facilitate installation and quantitative calculation. The sampling frequency of the magnetic sensor is 200 Hz, as shown in Figure 2, and its control core is the microprocessor MCU STM32F103C8T6 (ST, IT), which uses the SPI (Serial Peripheral Interface) communication protocol to read data in multiple channels. The main control module integrates the USB to TTL reading module, and the data can be transferred to the PC by using the USART (Universal Synchronous/Asynchronous Receiver/Transmitter). The system is powered by a 3.3 V DC power supply to ensure the normal operation of all components.

**Figure 3:**
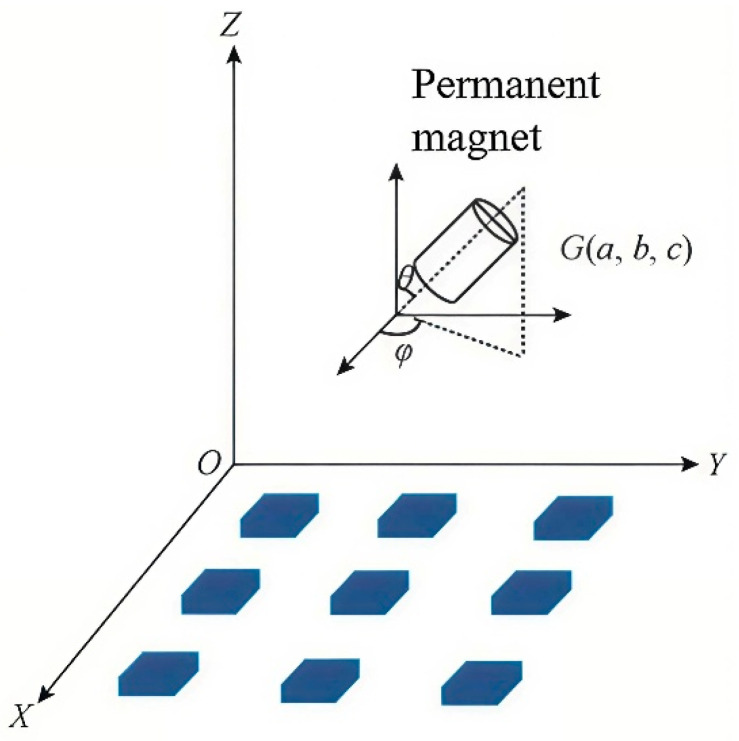
Schematic diagram of magnetic positioning.

The magnetic positioning is a technique that determines the spatial position of a permanent magnet based on the magnetic dipole principle, as illustrated in Figure 3.

Assuming the components of $H_{0}$ along the X, Y, and Z axes are (m, n, p), satisfying m^2^ + n^2^ + p^2^ = 1, and G(a, b, c) represents the center position of the magnet, while Q(xₗ, yₗ, zₗ) denotes the coordinates of any point in space. Let $R_{l}$ be the distance vector from any point Q(xₗ, yₗ, zₗ) to the magnet position G(a, b, c). Then the magnetic flux density at point Q can be calculated by Equation (1):

(1) $$B=B_{T}\left(\frac{3\left(H_{0}\cdot R_{l}\right)R_{l}}{|R_{l}{|}^{5}}-\frac{H_{0}}{|R_{l}{|}^{3}}\right)$$

Here, the constant $B_{T}=\frac{\mu_{r}\mu_{0}\pi b^{2}LM_{0}}{4\pi }$, where $\mu_{r}$ is the relative permeability of the medium and $\mu_{0}$ is the vacuum permeability. The three-axis magnetic field intensity can then be expressed as:

(2) $$\left\{\begin{array}{c} B_{lx}=B_{T}\left\{\frac{3\left[m\left(x_{l}-a\right)+n\left(y_{l}-b\right)+p\left(z_{l}-c\right)\right]\left(x_{l}-a\right)}{R_{l}^{5}}-\frac{m}{R_{l}^{3}}\right\}\\ B_{ly}=B_{T}\left\{\frac{3\left[m\left(x_{l}-a\right)+n\left(y_{l}-b\right)+p\left(z_{l}-c\right)\right]\left(y_{l}-b\right)}{R_{l}^{5}}-\frac{n}{R_{l}^{3}}\right\}\\ B_{lz}=B_{T}\left\{\frac{3\left[m\left(x_{l}-a\right)+n\left(y_{l}-b\right)+p\left(z_{l}-c\right)\right]\left(z_{l}-c\right)}{R_{l}^{5}}-\frac{p}{R_{l}^{3}}\right\} \end{array}\right.$$

(3) $$R_{l}=\sqrt{{\left(x_{l}-a\right)}^{2}+{\left(y_{l}-b\right)}^{2}+{\left(z_{l}-c\right)}^{2}}$$

For the magnet itself, there are six variables to be determined: its position G(a, b, c) and the normalized direction vector $H_{0}$ (m, n, p). In order to calculate the position of the magnet, a nonlinear algorithm is usually adopted for solution. Define the pose parameter vector X = (a, b, c, m, n, p)^T^, and construct the error function as shown in Equation (4):

(4) $$E\left(X\right)=\sum_{l=1}^{L}{∥B_{l}^{\mathrm{meas}}-B_{l}^{\mathrm{model}}\left(X\right)∥}^{2}$$

Here, l=1,2,…,N represents the number of sensors, with N=9 in this setup. $B_{l}^{\mathrm{meas}}$ is the actual measurement, while $B_{l}^{\mathrm{model}}$ is the calculation result from the model.

To improve localization accuracy, a weighted least-squares formulation is adopted:

(5) $$F_{w}\left(X\right)=\sum_{l=1}^{9}w_{l}∥e_{l}\left(X\right){∥}^{2}$$

where the weight $w_{l}$ is inversely proportional to the distance between the sensor and the magnet, i.e., $w_{l}=\frac{1/R_{l}}{\sum_{i=1}^{9}1/R_{i}}$, giving higher influence to sensors closer to the target.

This leads to a six-dimensional nonlinear optimization problem:

(6) $$X^{*}=\arg\underset{X}{\min F_{w}}\left(X\right)$$

To solve this efficiently, this study proposes a hybrid optimization strategy that integrates Particle Swarm Optimization (PSO) with a gradient-based local refinement method, followed by an Improved Differential Evolution (DE) algorithm and Extended Kalman Filter (EKF) for dynamic tracking.

Specifically, PSO is first employed for coarse global search. The PSO parameters are set as follows: population size of 300, inertia weight linearly decreasing from 0.9 to 0.4, and acceleration coefficients $c_{1}=c_{2}=2.0$. These values were empirically optimized through preliminary calibration experiments to ensure a balance between exploration and convergence speed. The PSO output serves as the initial guess for the subsequent Improved DE algorithm.

The Improved DE algorithm introduces adaptive parameter control and a hybrid mutation strategy. The mutation operation is divided into three stages based on iteration progress (early, middle, and late), each employing a distinct strategy:

(7) $$V_{i}=\left\{\begin{array}{cc} X_{r1}+F\cdot \left(X_{r2}-X_{r3}\right) & \left(\mathrm{early}\;\mathrm{stage}\right)\\ X_{i}+F\cdot \left(X_{\mathrm{best}}-X_{i}\right)+F\cdot \left(X_{r1}-X_{r2}\right) & \left(\mathrm{middle}\;\mathrm{stage}\right)\\ X_{\mathrm{best}}+F\cdot \left(X_{r1}-X_{r2}\right)+F\cdot \left(X_{r3}-X_{r4}\right) & \left(\mathrm{late}\;\mathrm{stage}\right) \end{array}\right.$$

The scaling factor F is adaptively adjusted according to:

(8) $$F=F_{min}+\left(F_{max}-F_{min}\right)\cdot e^{-\lambda \cdot \frac{g}{G}}$$

where g is the current generation, G is the maximum generation, and λ is the decay coefficient. This allows for larger F in early generations to promote global exploration and smaller F in later generations to enhance local exploitation.

The crossover probability CR is also adaptively tuned based on population diversity:

(9) $$CR=CR_{min}+\left(CR_{max}-CR_{min}\right)\cdot \frac{\bar{E}-E_{min}}{E_{max}-E_{min}}$$

**Figure 4:**
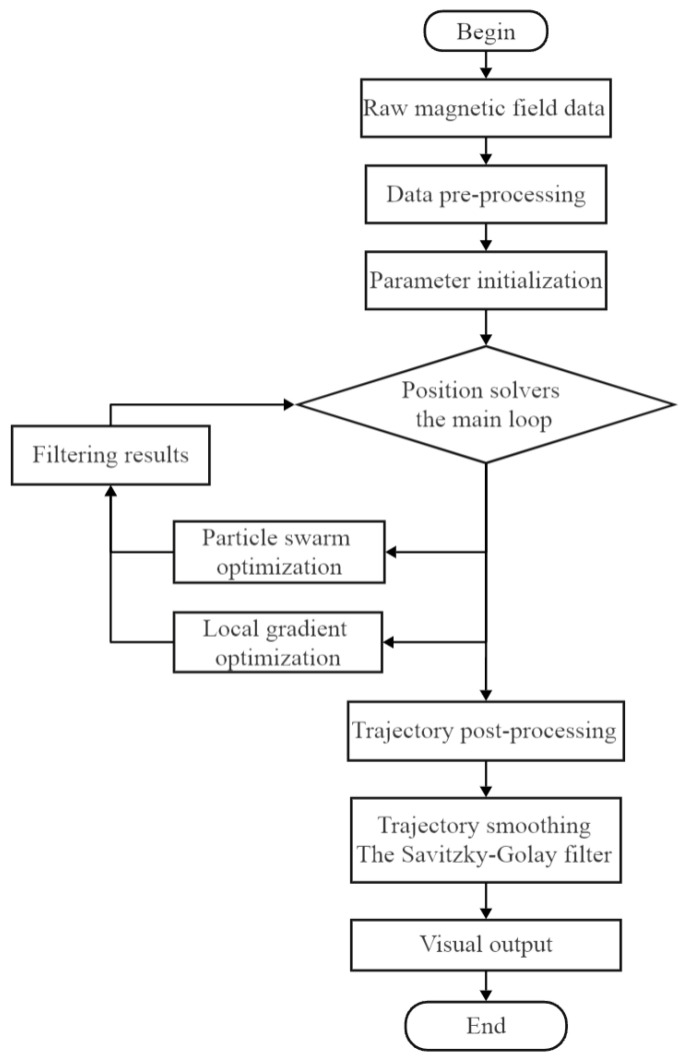
Position resolution process.

After improving the output position estimation of DE, the EKF is introduced for temporal fusion. The output of DE is taken as the observation value of EKF, and then a smooth and continuous trajectory is generated through the prediction and update steps. A block diagram of the algorithm is shown in Figure 4.

### 2.3. Validation of Magnetic Positioning Technology

**Figure 5:**
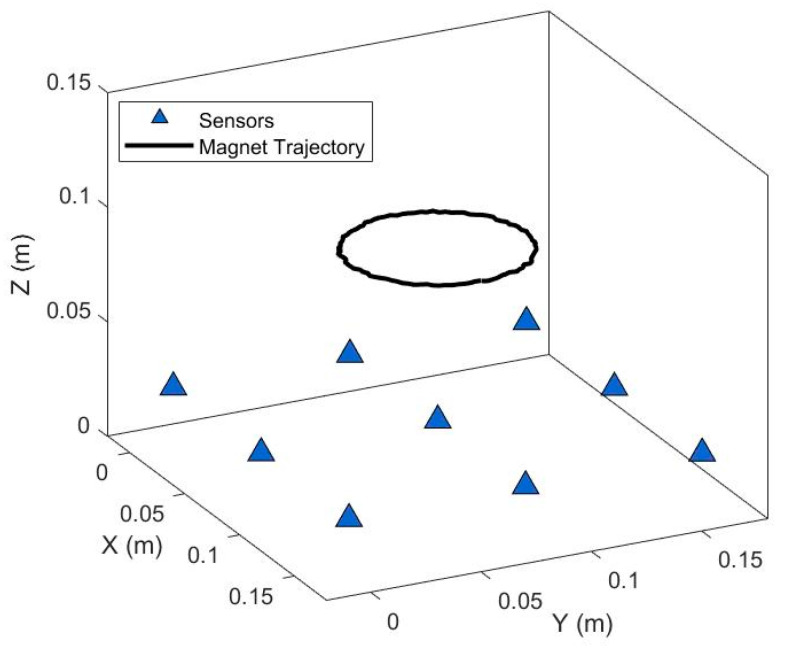
Curvilinear Motion Trajectory.

Circular motion was utilized to validate the dynamic trajectory of the magnetic positioning system. To be consistent with the experimental background, spherical permanent magnets with a diameter of 3 mm were selected for the experiment. The magnet was fixed on a plane 100 mm away from the measuring device and made to undergo circular motion with a radius of 40 mm around a fixed center (the central sensor). The positioning trajectory is shown in Figure 5. The *Z*-axis coordinate remained within the range of 99–101 mm, by comparing and calculating based on the known circular motion, the errors in the x and y axes were found to be maintained within the range of 0–2 mm.

**Figure 6:**
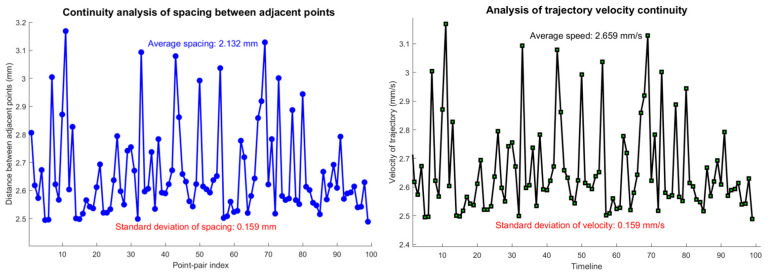
Trajectory continuity verification.

To further evaluate the continuity and smoothness of the system in dynamic trajectory, a continuity validation analysis was performed on the aforementioned circular trajectory. Trajectory continuity serves as a critical indicator for assessing dynamic positioning quality, directly influencing the system’s dynamic accuracy and stability in practical applications. By calculating parameters such as the distance uniformity between adjacent sampling points, motion speed consistency, and trajectory closure accuracy, the dynamic tracking performance of the system was quantitatively evaluated. As shown in Figure 6, the analysis results indicate that the standard deviation of the distance between adjacent trajectory points was 0.15 mm, the speed fluctuation coefficient was below 5%, and the closure error between the trajectory’s start and end points was less than 1 mm. This verifies that the system exhibits good continuity and smoothness during dynamic motion, meeting the requirements for high-precision magnetic positioning applications.

To characterize the performance of the proposed magnetic localization system, a series of localization experiments was conducted. The experimental results indicate that the system achieves a maximum dynamic localization error of 2.5 ± 0.5 mm and a maximum static localization error of 1.5 ± 0.5 mm. These error metrics are valid within a sensing volume of 30 cm × 30 cm × 25 cm, which is defined with respect to the central sensor of the array as the origin of the bottom reference plane. Compared to other positioning algorithms in magnetic localization [33], the proposed method achieves dynamic tracking while maintaining comparable static error. When compared with existing dynamic positioning methods [34], this study employs a greater number of sensors, reducing the positioning error from approximately 8.3 mm to 3 mm. This improvement effectively enhances positioning accuracy at the cost of a slower computational process.

The performance parameters of the aforementioned magnetic positioning system are summarized in Table 2.

## 3. Results and Discussion

The motion of particles within the rotating drum, particularly the behavior of high-density intruder particles relative to the ambient particles, is key to understanding the system’s movement mechanisms. The spatiotemporal distribution of their trajectories reflects the resultant force distribution. This study focuses on the macroscopic morphological changes in intruder particle trajectories and, combined with trajectory quantification metrics and flow layer status, systematically elucidates the evolution of dominant mechanisms and their stage characteristics during the process of increasing rotation speed.

### 3.1. Intruder Particle Trajectory Analysis and Quantification

**Figure 7:**
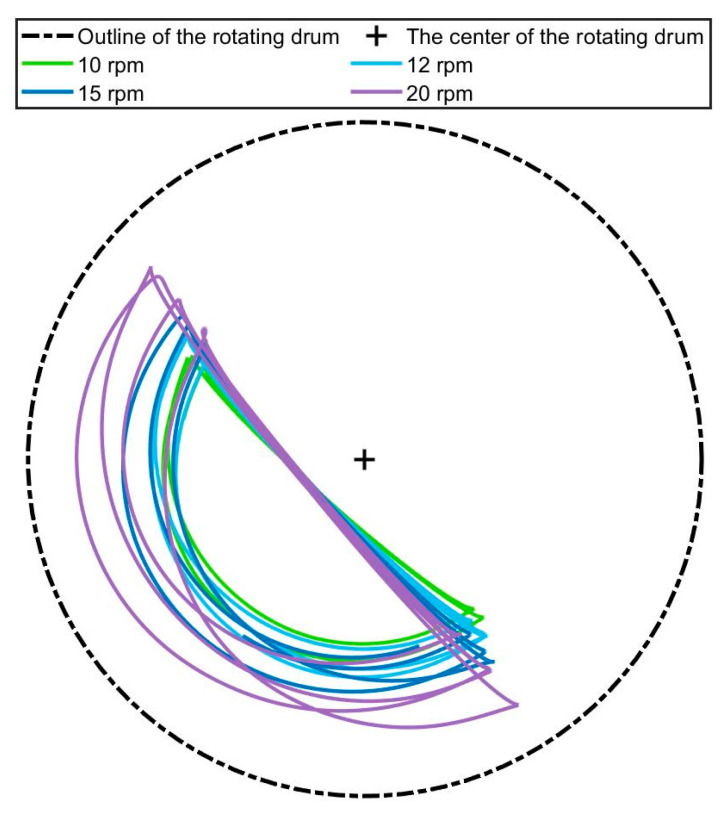
Motion trajectory of the permanent magnet.

In each experiment, the magnetic bead was placed at the same initial position in the rotating disk, with the rotation speed sequentially set to 10 rpm, 12 rpm, 15 rpm, and 20 rpm. Data from the interval starting from the initiation of motion until trajectory stabilization were extracted, as shown in Figure 7.

As observed in Figure 7, with the rotational speed increasing from 10 rpm to 20 rpm, the particle trajectories progressively shift from a region near the center toward the wall, exhibiting pronounced divergence at 20 rpm. This behavior suggests the emergence of competitive interactions among particle segregation mechanisms with increasing speed. To quantitatively characterize the dispersion of trajectories, we define the standard deviation of the radial distance of the particle position from the drum center, denoted as $\sigma_{r}$. In this axisymmetric system, radial distance serves as a natural coordinate, and σᵣ effectively quantifies the extent of radial mixing or segregation. Compared to alternative metrics such as the mean radial position or the range, σᵣ provides a more statistically robust measure of dispersion while remaining sensitive to subtle trajectory fluctuations. It is important to note that all raw trajectory data undergo rigorous preprocessing, including Kalman filtering and velocity-guided anomaly detection. As a result, σᵣ is computed from a cleaned dataset and reliably reflects the collective behavior of the particles. Although σᵣ alone may not fully capture every aspect of segregation competition, it serves as a valuable indicator for identifying regime transitions when integrated with analyses of flowing layer thickness and shear rates, as expressed in Equation (10).

(10) $$\sigma_{r}=\sqrt{\frac{1}{N-1}\sum_{i=1}^{N}{\left(r_{i}-\bar{r}\right)}^{2}}$$

**Figure 8:**
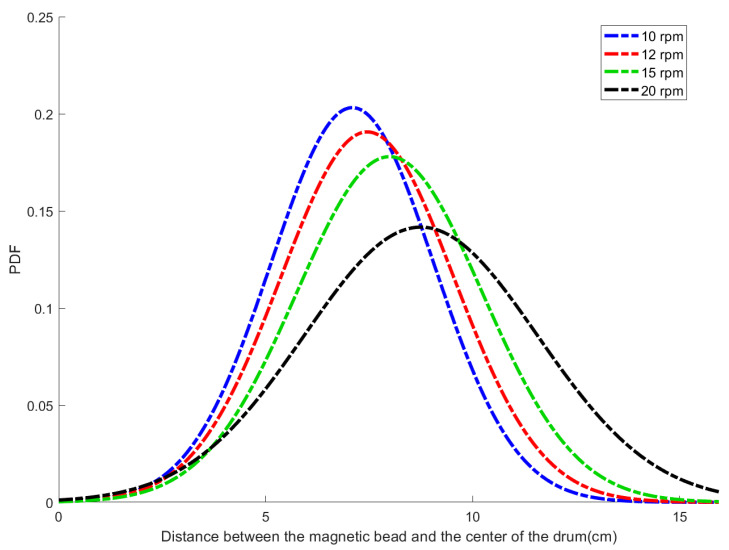
Distribution of trajectory distances from the center.

Here, N represents the number of trajectory points, $r_{i}$ denotes the distance from the i-th trajectory point to the center of the rotating drum, and $\overset{-}{r}$ is the mean radial distance. The statistical distribution characteristics of the distance between the magnetic bead and the drum center under different rotational speeds, fitted to a normal distribution, are shown in Figure 8.

Figure 8 illustrates the probability density distribution of the distances from the magnetic bead to the drum center under different rotational speeds. It can be observed that as the rotational speed increases from 10 rpm to 20 rpm, both the mean distance of the trajectories from the center and the distribution width gradually increase. This indicates an expansion in the fluctuation range of the particle trajectories along the radial direction, corresponding to an increase in the $\sigma_{r}$ value. Consequently, the dispersion degree of the trajectories rises.

### 3.2. Influence of Rotational Speed on Intruder Particle Motion Mechanisms

**Figure 9:**
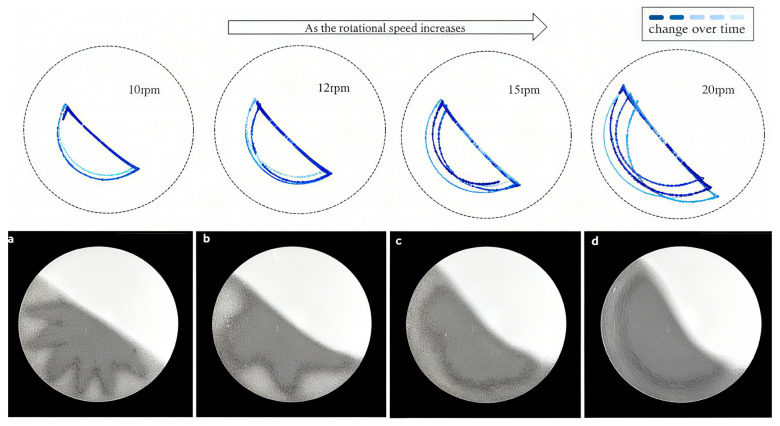
Particle motion under different rotational speeds; (**a**) 10 rpm; (**b**) 12 rpm; (**c**) 15 rpm; (**d**) 20 rpm.

Trajectories of the intruder particle were captured after the system reached relative stability, as shown in Figure 9. From left to right, the rotational speeds are 10 rpm, 12 rpm, 15 rpm, and 20 rpm, respectively. The trajectory color transitions from dark blue to light blue over the particle’s motion time.

Precise capture of the intruder particle’s trajectory in Figure 9 reveals a significant transition in the dynamical behavior of the particle system once the rotational speed exceeds a critical value of 15 rpm. As the speed increases, the trajectories shift from concentrated to divergent, with increased radial fluctuations, indicating the activation of competitive behavior among segregation mechanisms within the granular flow. In the corresponding pattern, the boundaries of particle distribution gradually become more uniform. At this stage, strong centrifugal forces not only promote macroscopic separation by density but also trigger complex, non-equilibrium interactions between particles. Interestingly, experiments by Jain et al. showed that particles approach nearly complete segregation at elevated speeds (up to 16 rpm) [16]. Our results further reveal intense dynamic instability within the system before the final segregated state is achieved, where reinforced competitive segregation mechanisms drive the evolution from a mixed distribution toward an efficient segregated state. This phenomenon suggests that particle segregation in a two-dimensional rotating drum is not a monotonic, smooth process but rather the result of multiple mechanisms (such as percolation, convection, and collision) competing for dominance. The critical behavior is closely related to specific operational conditions (e.g., rotational speed, particle properties).

**Figure 10:**
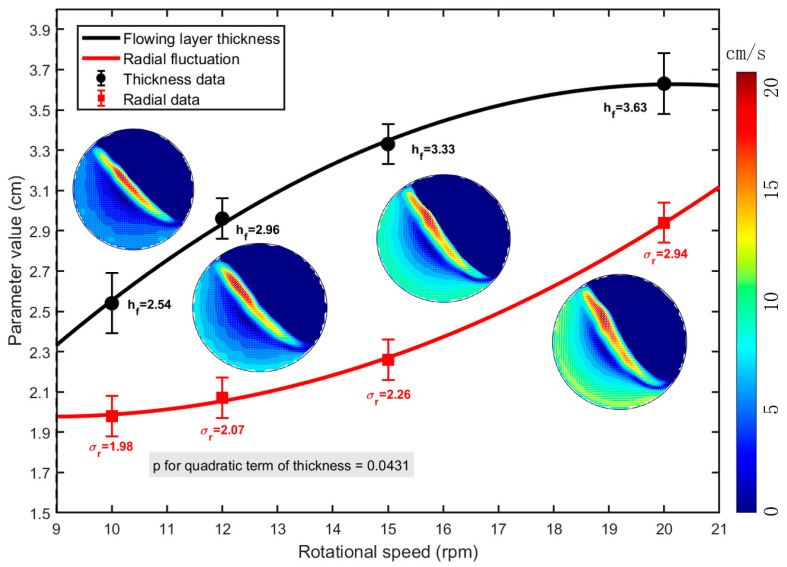
Relationship between kinematic parameters and rotational speed.

Typically, granular flow accomplishes mass transport within the flowing layer, where segregation also occurs. The thickness of this flowing layer exhibits a significant correlation with rotational speed [35]. The flowing layer thickness was determined using a high-speed camera combined with image processing. For each rotational speed, after the flow reached steady state, at least 10 s of images were continuously recorded. The free surface and the interface between the flowing and passive layers were identified based on velocity gradients. The vertical distance between these boundaries was measured and averaged at multiple radial positions in each frame to obtain the thickness. We established the relationship between rotational speed, flowing layer thickness, and radial fluctuation, as shown in Figure 10, where error bars represent the standard deviation from three repeated experiments. At the same time, we added the contour map of the velocity field under four characteristic rotational speeds to the figure.

Figure 10 indicates that at a drum speed of 10 rpm, intruder particle motion is relatively regular. The flowing layer is thin, and the particle’s range of motion within it is limited, constraining its entry position into the passive layer and resulting in minimal variation in its entry point into the flowing layer. As rotational speed increases, the flowing layer thickens, providing the intruder particle with greater vertical movement space. This allows the particle to traverse different velocity sub-layers, altering its entry position into the passive layer and directly changing its force environment. If located in a high-speed region, the particle migrates outward under strong inertial forces; if in a low-speed region, it is more susceptible to gravitational influence. This process essentially represents competition between percolation and diffusion dominated by inertial forces. This position dependence causes trajectories to gradually diverge with increasing speed, becoming unstable at 20 rpm.

Figure 10 also shows that the flowing layer thickness and radial fluctuation exhibit different growth trends: the gradient of flowing layer thickness with respect to speed gradually decreases, while that of intruder particle radial fluctuation increases. Statistical analysis confirms that the decreasing gradient of flowing layer thickness is statistically significant (*p* < 0.05 for the quadratic term), indicating that the growth rate of the flowing layer slows down as the particle bed approaches centrifugal equilibrium. We attribute these opposing trends to their governance by different dynamical mechanisms: the former’s reduced growth gradient stems from the particle bed approaching centrifugal equilibrium, whereas the latter’s increased gradient arises from amplified kinetic differences among particles at higher speeds. This discrepancy reveals a transition in the dominant mechanism as speed increases, which can be divided into three stages: gravity-controlled cooperative motion, collision-dominated local dispersion, and inertia-driven vigorous separation. In the initial stage, gravity–buoyancy balance enables cooperative flow with small radial fluctuations. As inertial forces strengthen, collisions become prominent, reducing trajectory overlap and increasing positional variation. Finally, inertial forces dominate, markedly accelerating the separation of high-density intruder particles and causing their trajectories to diverge drastically.

### 3.3. Transition Boundaries of Dominant Mechanisms

Figure 10 reveals that while the rate of change in flowing layer thickness gradually decreases, the radial fluctuation increases progressively with rotational speed. When the speed exceeds 12 rpm, the deceleration in flowing layer thickening indicates a transition toward a more fluidized state, where inertial forces progressively outweigh gravitational settling, whereas the sharp increase in radial fluctuation signifies the growing influence of collisions on positional variation—at this point, differences in the depth of particles within the flowing layer begin to dominate the divergence of their trajectories. Once the speed surpasses 15 rpm, the thickness change slows further while radial fluctuation continues to grow, marking the replacement of collision-dominated mechanisms by inertial forces. The position coordinate of the intruder particle within the centrifugal potential field then becomes the key determinant of its motion path.

As shown in Figure 10, the high-velocity regions become more pronounced and the overall velocity increases with rising rotational speed. The velocity distribution characteristics are intrinsically linked to the Froude number (Fr), which represents the relative magnitude of inertial to gravitational forces and provides a macroscopic quantitative standard for predicting changes in flowing layer thickness and particle motion trends. To clearly delineate the three stages of particle motion, the Froude number $Fr=\omega^{2}R/g$ is introduced to characterize the strength of inertia-dominated diffusion, and the shear rate $\dot{\gamma }=\partial u/\partial y$ is used to quantify gravity-dominated percolation, where ω is the angular velocity of the disk, R is the drum radius and is equal to 145 mm, and g is the gravitational acceleration. It should be noted that although the specific value of R linearly scales the Fr, the identification of regime boundaries is primarily based on observable transitions in particle kinematics rather than fixed threshold values, thereby ensuring the robustness of the relative stage sequence.

**Figure 11:**
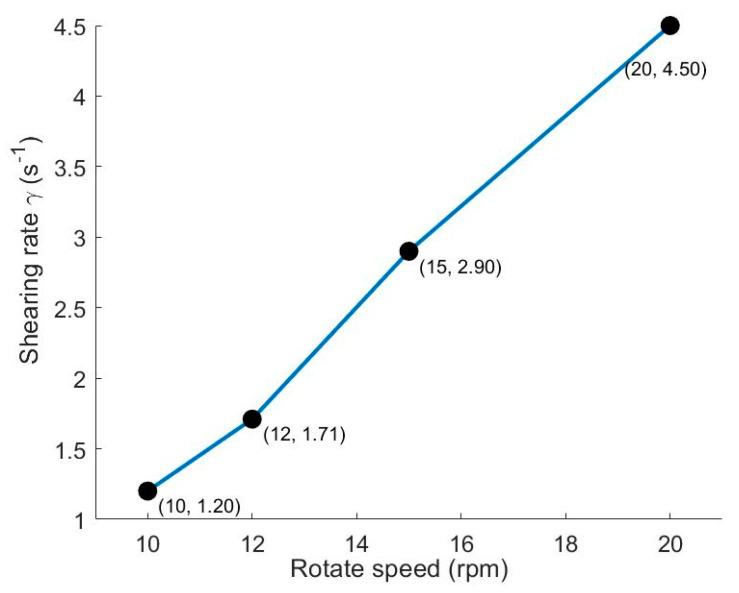
Shear rate at different rotational speeds.

The variation in the average shear rate with rotational speed is shown in Figure 11. A monotonically increasing nonlinear trend is clearly observed across the tested speed range (10–20 rpm). The shear rate increases relatively slowly as speed rises from 10 rpm to 12 rpm; its growth accelerates markedly within the 12–15 rpm interval, showing the most significant increase; beyond 15 rpm, the growth rate slows again, and the curve flattens. This pattern closely relates to the transition in particle motion states within specific speed ranges, with 12–15 rpm being the critical region where changes in the force mechanisms acting on particles trigger a sharp jump in shear rate. To clarify the regulatory role of shear effects on particle motion in the drum across different stages, a relationship is established with the Froude number as the horizontal coordinate and the shear rate as the vertical coordinate, analyzed in conjunction with the corresponding intruder particle trajectories at different speeds.

**Figure 12:**
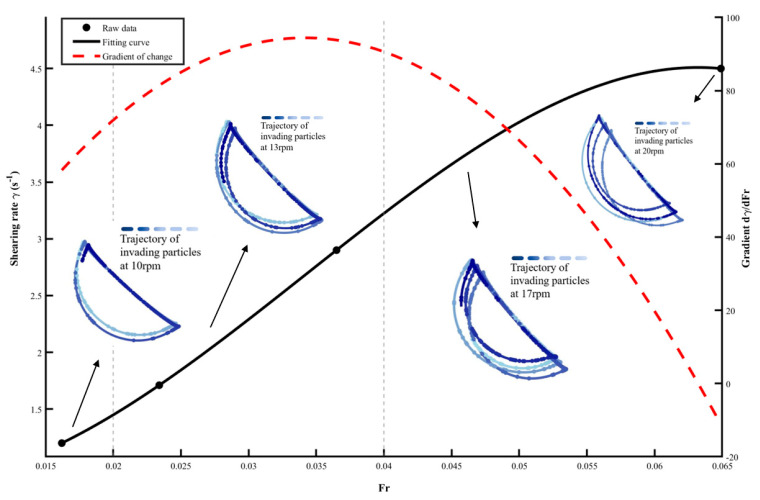
Evolution of Shear Rate with Froude Number and Trajectory Characteristics of Invading Particles at Different Rotation Speeds.

The black curve in Figure 12 shows a positive correlation between Fr and the shear rate. Changes in the slope of the fitted curve reflect transitions in the segregation mechanisms of the particle system, serving as an external manifestation of the staged transitions in the competitive relationship among gravity, contact stress, and inertial forces within the system. The red dashed line in Figure 12 represents the gradient $d\dot{\gamma }/d\mathrm{Fr}$, which quantifies the sensitivity of the shear rate to increasing inertial forcing. At low Fr, the steep slope indicates that small increments in centrifugal force sharply enhance the shear rate, reflecting a rapid intensification of collisional activity and kinetic stress gradients—key drivers of segregation. As Fr increases, the slope diminishes, suggesting that further increases in speed primarily affect particles via mean flow rather than proportionally amplifying the stress gradient. This nonlinear behavior thus captures the evolving balance between collision-driven diffusion and inertia-driven convection, providing insight into the transition of dominant segregation mechanisms.

Based on the above analysis, three typical stages of particle motion can be defined: the percolation-dominated stage, the mechanism competition stage, and the diffusion-dominated stage.

I. Density-driven Percolation Stage (ω < 12 rpm, Fr < 0.02)

In this stage, ambient particles are primarily constrained by gravity and contact stress. The thickness of the particle flowing layer grows relatively rapidly with system energy input, leading to an accelerated increase in shear rate. The motion of high-density intruder particles is confined by the ambient particles. Trajectory deviation is mainly driven by the overall flow field, increasing relatively slowly. Their greater inertia (due to density difference) causes a lagged response to the rapidly changing flow field of the ambient particles, resulting in long relaxation times, small trajectory fluctuations, and gentle changes.

II. Mechanism Competition Amplification Stage (12 rpm ≤ ω ≤ 15 rpm, 0.02 ≤ Fr ≤ 0.04)

In this stage, both percolation and diffusion motions intensify significantly, yet trajectories no longer converge fully. The high-velocity regions within the velocity field expand markedly, the radial fluctuation of intruder particles strengthens considerably, and the velocity gradient—i.e., the shear rate increases sharply, reflecting heightened velocity differentials within the particle bed. This pronounced enhancement of shear action promotes both the frequency and intensity of inelastic collisions between particles, and begins to effectively amplify the separation trend caused by inertial differences between the intruding high-density particles and the ambient low-density particles. As a result, intruder particles gain greater independence during collisions, their dispersion relative to ambient particles intensifies noticeably, and the radial fluctuation increases accordingly, exhibiting a strong positive correlation with the rise in shear rate within this interval.

III. Diffusion-Dominated Stage (15 rpm < ω ≤ 20 rpm, 0.04 < Fr)

In this stage, $\dot{\gamma }$ approaches its peak value, indicating that the system has entered a high-energy regime where both segregation and diffusion are intensified. However, the continued growth of radial fluctuation $\sigma_{r}$ suggests that diffusion becomes increasingly dominant in shaping the intruder’s trajectory, rather than implying that segregation has diminished or plateaued. As both segregation and diffusion scale with shear rate, their relative balance shifts: at elevated shear rates, enhanced velocity fluctuations amplify diffusive spreading, broadening the radial envelope while the inward drift driven by kinetic stress gradients persists. Inertial forces now dominate, causing intruder particles to migrate towards the drum wall under the action of streamwise inertial forces. This leads to a further significant increase in the radial fluctuation difference, reaching its maximum within the interval.

## 4. Conclusions

This study designed and constructed a high-precision magnetic array positioning system based on magnetic dipole theory, effectively overcoming the limitations of traditional measurement methods in terms of sampling rate and anti-interference capability. Experimental validation demonstrates that the system achieves a triaxial dynamic positioning error of less than 3 mm and trajectory continuity exceeding 99%, satisfying the requirements for high-precision motion tracking within complex granular systems.

Leveraging the periodicity and stability of granular flow in a quasi-two-dimensional rotating drum, a high-density magnetic bead was introduced as an intruder particle into a homogeneous bed of ambient particles. Using magnetic positioning technology, the influence of rotational speed on particle motion mechanisms was systematically investigated. Experimental results reveal three distinct stages in the motion of the intruder particle with increasing rotational speed: In the percolation-dominated stage (ω < 12 rpm, Fr < 0.02), particle motion is primarily constrained by the bulk flow field. The thickness of the flowing layer increases rapidly, leading to an elevated shear rate. However, due to its greater inertia, the intruder particle exhibits a delayed response, with smooth trajectory fluctuations and a prolonged relaxation time. In the mechanism competition stage (12 rpm ≤ ω ≤ 15 rpm, 0.02 ≤ Fr ≤ 0.04), both percolation and diffusion mechanisms intensify. Inelastic collisions between particles become more frequent, and motion separation induced by inertial differences begins to emerge. During this stage, the intruder particle gains increased motion independence, and its radial dispersion and trajectory divergence grow significantly with the rising shear rate. In the diffusion-dominated stage (15 rpm < ω ≤ 20 rpm, Fr > 0.04), inertial forces replace percolation as the dominant factor. Driven by strong inertial effects, the intruder particle migrates toward the drum wall, reaching its maximum radial fluctuation. The system transitions into a dynamic equilibrium state governed by inertial diffusion.

This research introduces a novel experimental method to observe particle motion in granular flows. The identified stage-wise characteristics enhance the understanding of segregation mechanisms and bridge macroscopic flow fields with microscopic particle dynamics. By establishing a baseline understanding of competing segregation mechanisms in a simplified 2D dry granular system, this work offers a mechanical analogy for interpreting complex geophysical flows like debris flows while emphasizing the need for further study under more realistic 3D and fluid-present conditions.

## Figures and Tables

**Table 1:** Parameters of the experimental setup.

Device	Parameter	Value
Particle	Diameter	3 mm
	Density	2500 kg/m^3^ (glass beads)
		7500 kg/m^3^ (magnetic beads)
Turntable	Diameter	290 mm
	Thickness	10 mm
	Rotating speed	10–20 rpm
	Fill degree	50%
Camera	Exposure time	1000 μs
	Acquisition frequency	300 fps
	Resolution	1280 × 1080
Magnetic positioning	Acquisition frequency	200 Hz
	Baud rate	115,200

**Table 2:** Magnetic Positioning System Performance Parameters.

Parameter Name	Parameter Value
Power Supply Voltage	3.3 V
Maximum Sampling Rate	200 Hz
Static Error	1.5 ± 0.5 mm
Dynamic Error	2.5 ± 0.5 mm

## Data Availability

The original contributions presented in this study are included in the article. Further inquiries can be directed to the corresponding author.

## Abbreviations

The following abbreviations are used in this manuscript:

SD	size and density
SPI	Serial Peripheral Interface
USART	Universal Synchronous/Asynchronous Receiver/Transmitter
PSO	Particle Swarm Optimization
DE	Differential Evolution
EKF	Extended Kalman Filter
Fr	Froude number
